# Software Techniques to Improve Data Reliability in Superconductor and Low-Resistance Measurements

**DOI:** 10.6028/jres.095.045

**Published:** 1990

**Authors:** L. F. Goodrich, A. N. Srivastava

**Affiliations:** National Institute of Standards and Technology, Boulder, CO 80303

**Keywords:** critical current, cryoconductor, editor, errors, figure of merit, measurement system, outlier, resistivity, software, statistics, superconductor

## Abstract

Software techniques have been developed to take low-amplitude data in various patterns, assign a figure of merit to a set of data readings, edit data for erroneous readings (or other experimental variations), and to alert the experimenter if the detected errors are beyond the scope of the software. Erroneous voltage readings from digital voltmeters, intermittent electrical connections, and an array of similar variations in data have been detected through the use of a data editor. The fixed-limit data editor removes readings that are inconsistent with the distribution of the majority of the data readings. The frequency of erroneous readings from a particular digital voltmeter ranges from 1 error per 100 000 readings to 1 error per 100 readings. The magnitude of the error can be as large as 3% of full scale with a zero volt input to the voltmeter. It may be necessary to have multiple meters measuring voltages in the same circuit in order to generate these erroneous readings. A systematic study was performed on the occurrence of the internally-generated erroneous voltmeter readings, and it was determined that the amount that a reading was in error scaled with one of a few parameters. The software techniques described here have been used in a variety of measurements, such as resistance-versus-temperature measurements made on cryoconductors or superconductors, and voltage-versus-current measurements made on superconductors to determine the critical current.

## 1. Introduction

In this age of computerized data acquisition, a computer and digital voltmeter^1^ are found in most modern measurement systems. These innovations have removed some of the subjective screening of data (that is, the determination of the validity of a set of data readings) which took place earlier with little forethought. Computers have also made the collection of vast amounts of data possible in a short period of time. While this is often desirable, the computer may give a number with as many digits as desired, with unknown significance. Thus, it is conceivable that the numerical output of a computer could be a mixture of both erroneous and correct data. Objective screening is difficult to introduce into software because of the variety of circumstances under which data acquisition takes place. Also, different levels of variation of the data readings tend to complicate the software. Since implementing screening software of this type is rather complicated, there is always the chance that the software will create errors. Therefore, a simple and expedient method to screen data is needed to improve data reliability.

In order to obtain reliable data, hardware tests of software techniques have been developed. These tests can be used to determine the integrity of both the software techniques and the hardware components of a measurement system [1]. One test that may be used to judge the performance of the software and the hardware components of a measurement system is the ability of the system to measure a null (zero) voltage. This is often quite challenging when voltage measurements are being made on superconductor samples. In this case, the ability of the system to measure zero volts is essential to obtain realistic and reliable data.

Another test that would investigate different aspects of the measurement system can be performed with the use of a dummy sample with finite resistance. In some cases, the system may measure a null voltage satisfactorily, but it may not measure a low-amplitude signal accurately. Null and finite resistance tests have proven to be invaluable in determining the integrity of the software and hardware components of a measurement system against thermal electric noise, common mode, random occurrences, and ground loop voltages.

Time constraints are often imposed upon the experimenter. These constraints may be present due to a combination of monetary and experimental problems. There are many relevant time constants of an experiment, such as the time needed to take a single reading from a meter, the thermal time constant, the system settling time, and the system drift time. Data acquisition would be a relatively simple task if time constraints of this type were not problematic. Since experimental constraints are usually not at nominal levels, efficient time management is of utmost importance.

The techniques described here have been useful in several types of low voltage measurements [2,3,4,5]. For example, they have been used to detect such experimental events as erroneous voltage readings, intermittent electrical connections, sample motion in a magnetic field, rapid changes in temperature, changes near a temperature transition on a superconductor, lack of thermal equilibrium, periodic and random electrical noise, and movements of humans near a sensitive experiment.

The software is designed to alert the operator (either through audio or visual means) if a problem is detected during data acquisition, thus making it easier to deduce the source of the problem and implement a solution. The software can also be designed to flag data for future editing, or in some cases, throw it away and attempt to take data again. In some cases, for example near temperature transitions, it is beneficial to store all of the measurements since data subsequently acquired may not have a smaller variation. It may also happen that the readings that were thrown away were the most interesting. In other cases, it may be necessary to perform repeat determinations until the variation of these data lies within the preset limits. In general, readings should not be eliminated from a data set without due consideration. Eliminating valid readings will affect uncertainty calculations.

The reliability of an experiment can be significantly affected by the pattern in which data is acquired. For example, in resistance-versus temperature-experiments, sample temperatures and transient settling times should be taken into consideration when determining the pattern in which data is to be acquired. Experiments have been performed in order to measure resistance as a function of temperature with transport dc current on superconductors, copper, copper alloy, and high strength, high conductivity metals. In these experiments, a forward-reverse-forward current data pattern was used. The pattern in which data is acquired can significantly change from experiment to experiment. For example, if voltage-current (*V-I*) measurements are being made on a superconductor, it is advantageous to create a data pattern generator which calculates current set-points that result in equally-spaced voltage readings on a logarithmic scale. This type of current distribution is necessary in order to fully characterize the extremely nonlinear voltage-current characteristics of a superconductor.

### 1.1 Terminology

Throughout this paper, a “reading” is defined to be a single measurement taken from a digital voltmeter. A set of readings is said to be “edited” if any erroneous readings have been removed from the set. A “data point” is a numerical compilation of a number of edited readings (an average of edited readings, for example), and a “data set” is simply a collection of related datapoints.

A set of “parallel readings” (as opposed to a set of consecutive readings) refer to a set of single readings that are taken in a sequential manner from different meters. For example, given three meters X, Y, and Z, a parallel reading pattern would be X(1), Y(1), Z(1); X(2), Y(2), Z(2); …; X(*n*), Y(*n*), Z(*n*), where *n* denotes the number of readings taken per meter. A set of “consecutive readings,” on the other hand, refer to a set of readings that are consecutively taken from different meters. In this case, the reading pattern would be X(1), X(2), …, X(*n*); Y(1), Y(2), …, Y(*n*); Z(1), Z(2), …, Z(*n*).

A “data run” (or simply a “run”) is a compilation of several data sets taken under various experimental conditions, for example, voltage readings at different temperatures, or critical current measurements at different magnetic fields. A post-run analysis may be desired to further analyze both the raw readings as well as the edited data sets.

A reading is said to be an “outlier” if it is inconsistent with the distribution of the majority of the readings. The difference between the correct reading and the outlying reading is called the “error.” A “data editor” is used to determine whether or not an outlier has occurred.

**Figure 1:**
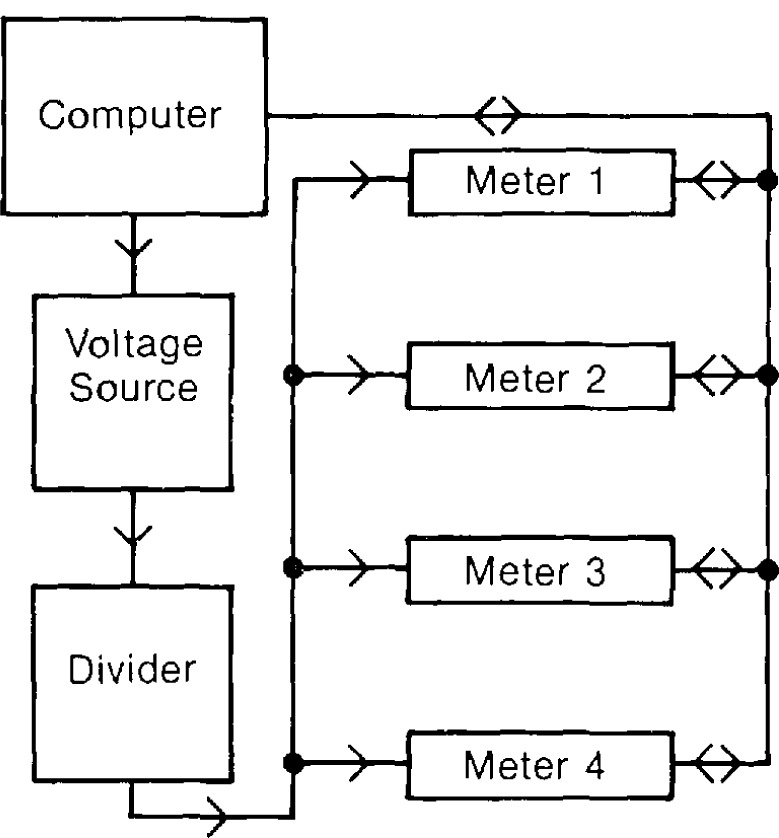
Block diagram of measurement system.

A “measurement system” consists of both hardware and software components. A block diagram of one of the measurement systems used in this experiment is shown in figure 1.

## 2. Software Techniques

The function of the software described in this paper is threefold. It can be used to assign a figure of merit to a particular data point, implement a particular pattern of data acquisition, and determine whether or not an outlier occurred. The software has been designed to provide feedback to the user (through audio and visual methods) in the event that an outlier occurred, or if the standard deviation of the majority of the readings is high. Software of this type is important not only for the determination of an occurrence of an erroneous voltage reading—it can also be used to correlate the experiment’s environment with an error in the data readings. It is necessary to design software editors to detect the presence of an outlier since outliers occur somewhat randomly, and are perhaps the most common source of errors in experimental work. Although the software discussed here is general in design, it has been empirically developed for superconductor and low-resistance measurements. More sophisticated statistical methods may be available [6], but the editor described here is simple, expedient, and sufficient for this type of measurement.

Given the set of time constraints under which many experiments are performed, it is prudent to do computations, generate a printout and a plot during settling times. The ratio of the time used to acquire data to the time needed for the measurement system to settle must be considered. It is inefficient to have a data acquisition time that is much less than the system settling time. A real time data editor must be simple enough so that the computation time is small, yet effective enough so that outliers and experimental variations in the data can be identified and the experimenter alerted.

### 2.1 Fixed Limit Editor

The fixed limit editor may be used for routine measurements under relatively low or known noise conditions. The editor limits would define an upper and lower bound for the acceptable variations in data readings.

After a collection of readings has been taken during an experiment, it is stored in matrix form on a permanent mass storage medium for future use (perhaps by a post-run analysis routine). One column of the reading matrix is extracted and stored in vector form. The readings in this vector are sorted from the smallest value to the largest value.

The preset editor limits are applied about the median of a sorted reading vector. Those readings that are within the upper and lower limits of the editor are used to calculate the average and the standard deviation of the edited readings. Any outliers that may be present in the readings appear either above the upper editor limit, or below the lower editor limit. If the number of readings outside the bounds of the editor is greater than the larger of two readings, or 3% of the total readings, the user should be alerted either through audio or visual means. If a similar number of readings is consistently found to be outside the bounds of the editor, the user should increase the allowed range for the readings, if they are consistent with the distribution of the majority of the readings.

Before the actual experiment is performed, a preliminary experiment should be conducted to determine a typical standard deviation using a large number of readings per data point. The preset editor limits should be five to ten times the standard deviation of these readings. In superconductor measurements, the extremely nonlinear voltage-current (*V-I*) characteristics can result in an increase in signal variation with voltage. In this case, two data points should be considered: zero current, and a current that results in a sample voltage that is near the typical maximum. A subsequent calculation of the standard deviation of the voltage readings would lead to appropriate editor limits. This preliminary experiment should be conducted periodically so that any changes in the measurement system can be accounted for in the editor limits. The time constants of the experiment can also be determined during this preliminary experiment. During the actual experiment, the number of readings that are taken per point could be reduced, so that the experiment can be performed more efficiently. During these measurements, it is important to monitor the relationship between the standard deviation and the editor limits to determine changes in noise conditions.

### 2.2 Data Patterns and Figures of Merit

Depending upon the type of measurements being made, the *pattern* in which data are obtained may significantly affect the ability of the experimenter to analyze the data and perform any corrections that may be necessary. Thus, it is important to implement a pattern suitable to the type of experiment being performed [5]. This reduces the number of assumptions that the experimenter must make in the data analysis.

**Figure 2:**
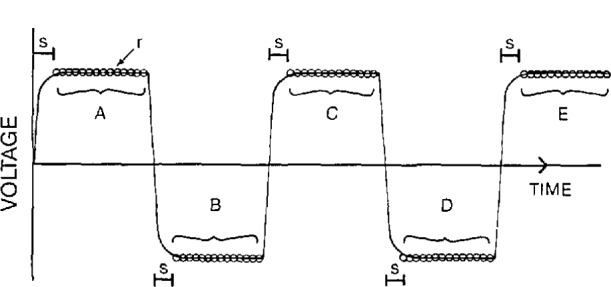
Data pattern of forward-reverse-forwared current.

In resistance-versus-temperature measurements on superconductors, copper, and copper alloys, a data pattern as depicted in figure 2 was incorporated in the software. A four-wire measurement with current reversals to correct for thermal-electric and offset voltages was used. This assumes that the current is constant and has a known magnitude. The software was designed to measure voltage, temperature, and time in parallel.

In the resistance-versus-temperature measurements, the data pattern of forward-reverse-forward current was used. In this data pattern, current is first injected into the sample in one direction (defined as the forward direction), and a number of voltage readings from the voltmeter are acquired on a computer. The current is reversed and an additional time is allotted for the settling of any transient effects in the system (region s in fig. 2). After the system has settled, voltage readings are obtained for the reverse current. The current is switched back to the forward direction and another fixed settling time is allotted for transient effects to decay. Finally, data corresponding to the forward current is obtained. This pattern could be continued or repeated after an experimental parameter has changed. For example, in the resistance-versus-temperature experiment, after the sample temperature changed by a certain amount, the forward-reverse current injection pattern was used.

In order to correct for any thermal electric effects that may be present, the two forward-current voltage readings (regions A and C, for example) are averaged to approximate the thermal effects that occurred at the approximate time of the reverse-current readings, region B in figure 2. The actual time of individual or groups of readings can be stored to avoid this approximation. This average is combined with the average of the reverse-current voltage data to give the effective voltage drop across the sample (assuming forward and reverse currents are identical in magnitude). The resulting equation for the data point value associated with regions A, B, and C is:

$$\text{data point}\left(\mathrm{ABC}\right)=\frac{1}{2}\left|\frac{\left(A+C\right)}{2}-B\right|$$ (1)

The difference between the two forward voltages can be assigned a figure of merit for the measurement, which can be used as a basis for editing or flagging individual printouts after the data run is complete. This sequence of forward and reverse current data points can be continued as necessary. The combination of any three adjacent data points would provide an associated figure of merit (regions A, B, C; B, C, D;…). The figure of merit can be calculated using the following equation:

$$\begin{array}{l} \text{Figure of Merit}\left(\mathrm{ABC}\right)\\ \;=\left|\mathrm{A-C}\right|/\left(\text{data point}\left(\mathrm{ABC}\right)\right), \end{array}$$ (2)

where data point (ABC) is calculated using eq (1). Thus, a forward-reverse data pattern configuration proved to be useful in the thermal electric corrections described above.

If the forward-reverse current data pattern is not feasible in the type of experiment being performed, it may be necessary to use a “zero-forward-zero” current data pattern. For example, if the experiment prohibits the application of a negative current (a diode in the circuit, for example), or if the magnitude of the reverse current is not identical to the magnitude of the forward current, then it is necessary to use a zero-forward-zero data pattern.

### 2.3 Example Experiment

**Figure 3:**
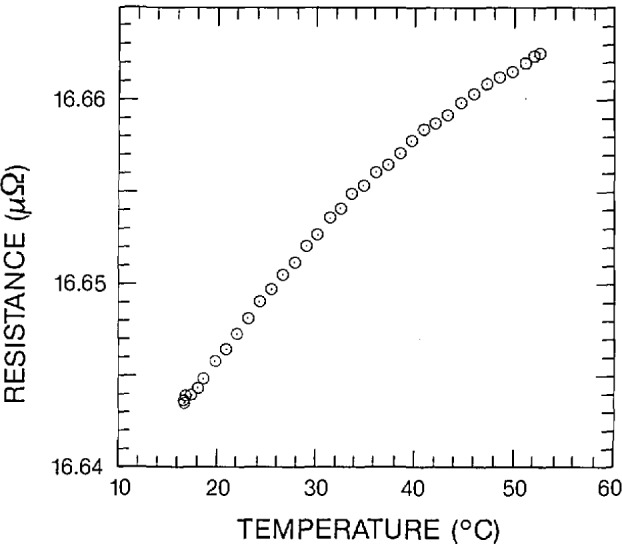
Shunt resistance as a function of temperature.

Several experiments have been performed using the software techniques described in the preceding section and the digital voltmeters discussed in section 3. As described in section 3, these voltmeters generate occasional erroneous readings. However, they are very suitable for superconductor and low resistance measurements. One experiment of this type was to determine the temperature dependence of a 16.6 *µ*Ω (6000 A) shunt resistor. The results of this experiment were to be used in critical current measurements of superconductors. In critical current measurements, the magnitude of the transport current, (the current flowing through the superconductor) must be accurately measured. The transport current was calculated from the voltage across a known shunt resistor. Since the temperature of the shunt resistor increases with increasing transport current, the temperature dependence of the shunt resistor must be precisely known. The software techniques discussed earlier resulted in precision on the order of a few thousandths of a percent. A plot of the shunt resistance as a function of temperature is presented on figure 3. A current of 500 A was used (which is low compared to the resistor’s 6000 A rating) in order to reduce the effect of self-heating of both resistors. A typical current drift was about 0.5 A, which is 0.1% of the total current.

**Figure 4:**
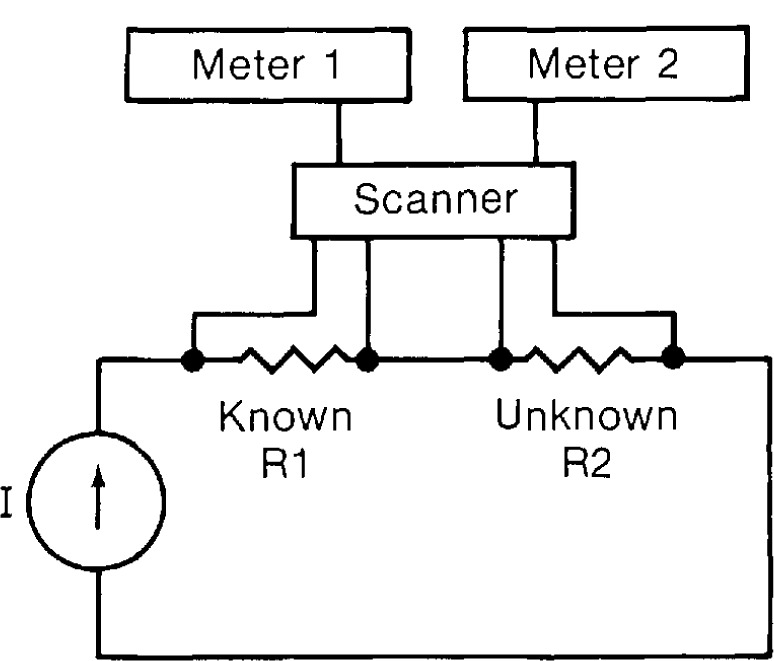
Block diagram of shunt resistor experiment.

In this experiment, the current supply, a known resistor, and the unknown resistor were connected in series, as is schematically shown on figure 4. Voltage leads from each resistor were fed into a scanner. Two digital voltmeters were also connected to the scanner. The scanner allowed each meter to measure either voltage signal. Precise voltage measurements and the calibrated shunt resistor can be used to determine the resistance of the unknown resistor. A data pattern of zero, forward, and zero current was used.

**Figure 5:**
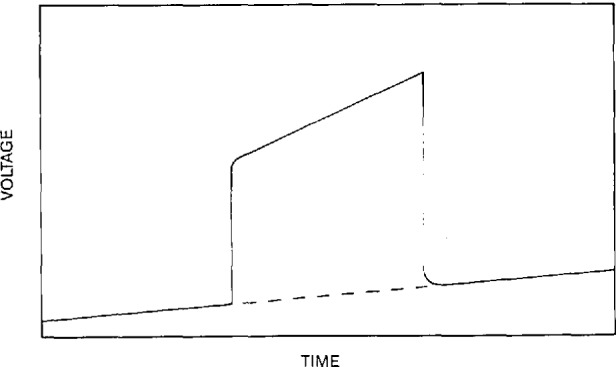
Data pattern of zero-forward-zero current with exaggerated current drift.

Frequently, the “forward” current has a small drift in time, as is schematically presented in figure 5. (The current drift has been exaggerated in this figure.) The drift in the forward-current regions is attributed to changes in the thermal electric voltage and drift in the current. The drift in the zero-current regions is attributed to changes in the thermal electric voltage. The forward current drift can be accounted for by using the data taken with zero current. This correction can be performed by calculating a linear voltage-time fit between the readings in zero-current regions, (as indicated in fig. 5 by the dashed line), and subtracting the resulting best fit line from the readings in the forward current region. A set of calculated instantaneous resistance values can be obtained by taking the ratio of the corrected voltage and current readings. The current readings are inferred by using the voltage drop across a standard resistor. The instantaneous resistance values can be edited to remove any erroneous data, and an average and standard deviation of the instantaneous resistance readings can be subsequently calculated.

If a leakage current is present in the nominally zero current regions of the pattern, then the unknown resistance is calculated using the ratio of the change in the voltage readings to the change in the current. This technique of calculating the instantaneous resistance is only applicable if the sample is ohmic. If the sample is non-ohmic, this calculation would result in a differential resistance. It may be possible to reduce the effect of leakage current by open-circuiting the system. However, this could change the effects of the circuit ground.

Voltmeters often have calibration errors associated with them. In order to approximately cancel this error, it is useful to use a scanner to interchange the voltage signals that are being fed to the voltmeters. The approximate cancellation of linear calibration errors only takes place if the deviation between the calibration constants of the two meters is small. Since another calibration error may exist between the voltmeter ranges on a given voltmeter, the two voltmeters were kept on the same voltage range. A single meter could be used to measure the voltage across both shunt resistors and reduce the inaccuracy in the experiment. However, one meter can be used only when the current and temperature are in steady-state conditions.

## 3. Erroneous Voltage Readings

The erroneous readings discussed here have provided a relevant and repeatable phenomenon that can be used to test the ability of software to discriminate erroneous from correct readings. This ability is necessary to detect experimental problems such as occasional noise spikes, human intervention, and transient conditions. If not detected, these types of experimental problems can lead to unreliable and generally unrepeatable readings. The fixed limit editor was used in this experiment to detect the presence of outliers.

This example is not intended to be a harsh labeling of digital voltmeters—it is only meant as a general caution on their use. They are clearly superior for obtaining automated, precise, and accurate measurements. If the digital voltmeters discussed here are used in conjunction with a software editor, it is not unreasonable to obtain precision on the order of a thousandths of a percent. The same model voltmeter described in this section was used to take the measurements in section 2.3.

### 3.1 Observation of Outliers

During voltage calibrations and resistance versus temperature measurements, it was observed that the digital voltmeters would occasionally give an erroneous reading. This phenomenon was first noted during attempts to reduce the variation of voltage readings being made during resistance-versus-temperature measurements. The voltmeter’s manufacturer stated in their Operator’s Manual that the meter occasionally exhibits “internally generated noise spikes.” Averaging more readings (20 to 50 readings) and using longer settling times between current reversals did little to alleviate this problem. It was also discovered that turning off the voltmeter’s signal filtering capability had a beneficial effect. The voltage readings were plotted using an automatic plotting routine. Occasionally, the limits of the voltage axis were significantly changed to accommodate what turned out to be outlying points.

### 3.2 Digital Voltmeter

The internal digital filter on this particular voltmeter switches on and off based on a comparison of the new reading and the accumulated running average. A scheme of this type is used so that if there is a rapid change in voltage readings, like a current reversal, the meter can quickly respond. However, the filter can generate a series of erroneous readings if a single outlying reading occurs. This results in a series of readings that approach the correct reading within arbitrarily small differences, thus making it difficult or impossible to edit the filtered readings. Also, using the internal digital filter creates some uncertainty with respect to the appropriate time to assign to a voltmeter reading. Editing filtered data is especially difficult when a few (10 to 50) readings are taken. Thus, it is beneficial to take data with the filter off, and edit the data using the computer before averaging. Using the filter when manually reading the meter display is advisable since it is difficult to average the displayed reading. If the filter is used in conjunction with a computer, the resultant readings will have an artificially low standard deviation.

### 3.3 Systematic Study of Erroneous Readings

The outliers were observed on one particular voltmeter model. Four meters of this model were tested extensively, and two other meters had limited testing. A voltage source and a resistive divider were used to generate an input signal which was split into four digital voltmeters. A block diagram of the system used is given in figure 1. The voltage divider in figure 1 was omitted for the larger signals. Each meter had the same input signal and should therefore measure the same value within calibration limits. An input voltage level was set and allowed to settle for about 10 min. A set of 1000 readings were taken in parallel from each of four meters. This allowed for an effective reading rate for each meter of 3.6 readings per second on the millivolt ranges, which is close to the ideal single meter reading rate of 4.0 readings per second. The effective rate was about 7.3 readings per second on the voltage ranges compared to the ideal reading rate of 8.0 readings per second. Thus, a total of 4000 readings were taken in 278 s on the millivolt ranges, and 137 s on the voltage ranges.

Next, the median reading was determined for each meter and the average and standard deviation calculated from the readings that were within preset limits of the respective median reading. A statistical summary of the readings on each meter and the location of any outlier that may have occurred were stored. If an outlier occurred during the data acquisition, a printout was made that included the average, standard deviation, and the maximum and minimum reading for each meter. The maximum and minimum readings were calculated after the outlying reading(s) was removed from the data point.

In order to obtain statistically significant results, a vast number of voltmeter readings had to be acquired, edited, and analyzed. In total, 2.4 million readings were taken for each data run, (there were 600 data sets consisting of 1000 readings taken from each of 4 meters) and more than 14 data runs were made. A few runs have been chosen for illustrative purposes here. Two of these runs depict the differences between the millivolt and volt ranges (in particular the 2-mV and 2-V ranges) of the voltmeters.

### 3.4 Data Plots

Three figures have been included here for each of the 2-V and 2-mV data sets to illustrate various characteristics of the outliers. The 2-V data set corresponds to the “a” figures and the 2-mV data set corresponds to the “b” figures. The number of outliers within each data set, along with a histogram of the spacing between adjacent outliers over all data sets has been displayed in figures 6a, 6b, 7a, and 7b. A statistical summary of the edited readings is presented in figures 8a and 8b.

Figures 6a and 6b illustrate the relative frequency of an outlier in a data set. Most data sets have no outliers. The majority of the outliers occur in data sets that contain multiple outliers. Therefore, the outliers seem to occur in bursts. The outliers on a particular meter seem to occur independently of outliers on other meters. For example, on figure 6a, the errors made by meters 1 and 2 were independent of each other in the sense that they did not occur in the same data set. If an error was present on all four meters at the same time, the error may be attributed to a variation in the input signal. A similar comparison of the other meters show that the errors are not correlated with a variation in input signal. There were only a few cases in which two of the four meters had adjacent outlying readings. Figure 6b clearly indicates the independence of the errors made by the meters. Near set 300, meter 1 had bursts of approximately 2 error readings per 1000 readings, while the other meters did not show any errors in those data sets. Figures 6a and 6b have similar overall characteristics, although the 2-V data set had more outliers than the 2-mV data set.

Figures 7a and 7b display a histogram of the separation between adjacent outliers within each of the 600 data sets. Most of the outliers within a data set are separated by at least 20 correct readings. This is relevant when considering the likelihood of a large number of outliers in a small data set. Thus, it is not likely that a set of 15 readings would contain a significant number of outliers.

Figures 8a and 8b display the distribution of the maximum and minimum values of the edited voltage readings about the average of the data set. The middle line in each plot indicates the standard deviation of the data sets. All of these values were computed after removing the outliers from the data set. Meter 1 on figure 8a shows a larger variation of voltage readings between sets 38 and 152. This increase may be attributed either to poor connections of voltmeter to voltage source or an increase in the internal noise of the meter. As can be seen from figure 8a, the increase in noise diminishes after data set 152. Although the variation in the signal was larger, the limits of the fixed limit data editor were such that most of the readings were not classified as outliers, as can be seen in figure 6a. Meter 2 has a different characteristic when compared to the other meters on figure 8b. However, meter 2 had the lowest frequency of outliers on the millivolt range. There were some relatively high minimum and maximum values on figure 8a and 8b that are attributed to random events which have magnitudes that were within the limits of the editor.

**Figure 9:**
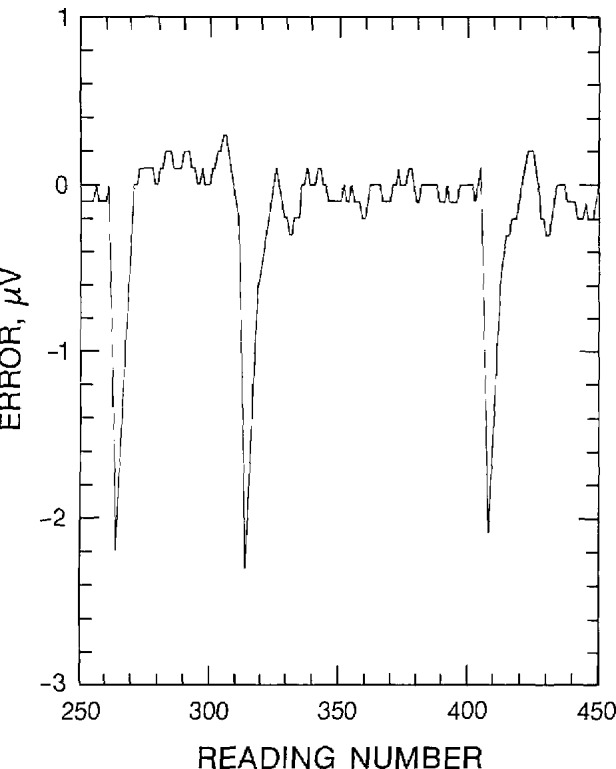
An occurrence of outliers with the internal digital filter on.

Figure 9 is included to illustrate the combined effect of the meter’s internal digital filter and the occurrence of an outlier. This graph displays the relative error (in *µ*V) as a function of the reading number. A single outlier probably occurred near the leading edge of each of the three sharp spikes. The meter’s internal digital filter may have switched off as it received the erroneous reading, but it did not switch off again after the erroneous reading. Consequently, the subsequent readings indicate an apparent integration or accumulating average that approaches the correct reading. These examples were somewhat atypical. More often, the filter switched off twice, creating an isolated point. In general, the frequency of the outliers was about the same with and without the digital filter. There were more errors with the filter on. These errors were most probably due to an occasional integration of a single error.

### 3.5 Data Tables

The observed errors scaled as a function of one of the following three parameters: the bias level (actual input signal), the selected voltage range of the voltmeter, and the difference between the maximum allowable value of the selected range and the input signal. In 99.07% of the cases, the error was a fixed percentage of one of these parameters (tables la through 2b). Tables 3a and 3b show the magnitude of the error caused by an outlier, along with the number of standard deviations the error is above the correct readings.

Each of the tables 1a through 2b contains the meter number, the type of error, (an error due to the bias level, voltage range, etc.) and the percentage category (0.6%, 3.0%, etc). The table entries are the actual percentage error, and the number of such errors is indicated in parentheses.

As mentioned earlier, the outliers were a fixed percentage of one of the above parameters. Out of the 9.6 million readings represented in these four tables, 2162 readings were outliers (approximately 1 in 4440 readings). Of the 2162 outliers, 2142 (99.07%) outliers can be characterized as an error due to either the bias level, the voltage range, or a difference in the voltage range and the bias level. Of the remaining 20 outliers, 3 can be attributed to the simultaneous occurrence of two outliers from the above categories. For example, one of these outliers could be categorized into a −2.4% of bias error. An outlier of this type can be attributed to a simultaneous occurrence of a +3.0% of bias error and a −0.6% of bias error. The remaining 17 outliers can be attributed to infrequent random events.

The errors listed above were infrequent when compared to the total number of readings that were taken from the voltmeter. However, after a single error occurred, the chance that more errors would occur on the same meter increased, as indicated in figures 6a and 6b. Thus, if an experiment’s time constraints dictate that a relatively small number of readings be taken per data point, the occurrence of one or more outliers in the readings could significantly affect the computed average. For example, on the highest voltage scale, a 3% of range error could result in an outlier with a 60-V magnitude. Thus, if the correct reading was 15 V, the outlying reading would be 75 V. As another example, if the actual reading is 1 *µ*V on the 2-mV range, a 3% of range error outlier would have a magnitude of 60 *µ*V, resulting in a 61-*µ*V erroneous reading. Erroneous readings of this type would significantly alter the unedited average.

A comparison of the error patterns on the 2-mV and the 20-mV range of the meters (with the same bias level) exhibits the different scaling of the bias and the range dependent errors. The 2-V and the 20-V ranges also exhibit a similar scaling of errors. There were some slight systematic differences between the percentage scaling of the errors for different meters. A similar pattern continued for the 200-mV and the 200-V ranges. The highest voltmeter range was 1000 V, but the errors scaled as though this range was 2000 V. Other data sets with the internal digital filter on showed the same scaling of errors.

The standard deviation of the outliers in each error category was approximately the same as the standard deviation of the allowed readings (see figs. 8a and 8b). For example, on the millivolt ranges of the meter, standard deviations of 27 nV and 28 nV were typical for the outliers and the allowed readings, respectively. Similarly, on the voltage ranges, standard deviations of 4 *µ*V for the outliers and standard deviations of 3 *µ*V were typical. The voltage difference between the correct readings and the outliers was significant compared to the standard deviation. For example, with a 0.6-mV bias on the 2-V range, the lowest error would be 3.6 *µ*V (the actual outlier reading would be 0.6036 mV, which is 0.6% of bias). Thus, the lowest error in this case would be 128 times the standard deviation of the correct readings.

Tables 3a and 3b show the error value and the number of standard deviations that the error is above the correct readings for the 2-mV and the 2-V ranges. Standard deviations of 28 nV and 3 *µ*V were typical for the correct readings for the 2-mV range and the 2-V range, respectively. Notice that most of the errors are hundreds or thousands of standard deviations above the correct readings. At these signal levels, these errors were clearly extreme outliers. At lower signal levels, the 0.6% of bias error can be difficult to identify.

### 3.6 Discussion of Erroneous Voltmeter Readings

The voltmeters were transmitting data to the computer via a standard IEEE bus cable. Since the errors were visually observed on the voltmeter’s display, it was deduced that the IEEE cable was not the source of these errors.

The regular patterns of the errors suggest that the source of the errors were not due to thermal electric effects. However, an effort was made to reduce the thermal effects by allowing for long settling times (between 10 and 60 min) between experiment setup and actual data acquisition. Also, leadshot bags were placed under and on top of the voltmeter lead connections in order to reduce thermal effects.

The manufacturer of the voltmeter is currently in the process of identifying the source of the erroneous readings. The manufacturer will also verify that this problem is limited to the situation in which multiple units of a particular model are used to measure voltages at the same time in an experiment. Erroneous readings have not been observed when only a single meter is used.

It is suggested that the outliers result from analog-to-digital conversion errors due to the discrete nature of the errors. In a typical experiment, multiple meters are used to measure different voltages in the same circuit. Even in this case, erroneous readings have been observed. The voltmeters that were used in these experiments are one of the most sensitive instruments presently available. Thus, they are probably pushing the present “state-of-the-art” in voltage measurements.

## 4. General Discussion

Although excellent commercial software packages for data acquisition may be available to experimenters, often they cannot be modified for specialized measurements. Invariably, due to the specialized nature of many experimental setups, data acquisition software must be designed by the experimenter. As is true for any sophisticated computer program, the experimenter must fully test the software under various conditions before proceeding further. Otherwise, it may be possible that the software either generates errors, or allows erroneous measurements in the system.

The editor discussed in this paper has been developed to remove those readings that are inconsistent with the distribution of the majority of the readings during data acquisition. The editor is designed to warn the user if the standard deviation of the edited readings is high, or if the number of points removed by the editor is greater than the larger of two readings or 3% of the total number of readings. These tolerances are a function of the inherent noise of the measurement system, the sensitivity of the measurement system, and the number of readings in a data set. If the editor warns the user that the number of discarded readings exceeds the tolerance, the user can investigate the source of the errors and determine a method to rectify the problem. There may be experiments in which a tolerance of 1% or less is appropriate. In this case, it will be necessary to adjust the editor so that the operator is not desensitized by being frequently alerted. Although the editor may eliminate some data from calculations during data acquisition, the software routine should be designed to store all of the data for future analysis using more sophisticated techniques.

It is necessary to use some type of outlier rejection rule [6] when analyzing data taken when outliers may be present. The rejection rule that best suits an experiment may be affected by the details of the measurement system, especially under low signal-to-noise ratio conditions. The fixed-limit editor described here may not be optimum; however it is clearly better than not using an editor. The editor limits can be adjusted so that only extreme outliers are rejected.

## 5. Conclusions

A software routine can create a data pattern, assign a Figure of merit to a data point, compute edited averages and standard deviations, and store the analyzed data. A summary printout of the data taken during a particular experiment also proves to be beneficial. It is wise to store as much raw (unedited) data as possible, so that it can be analyzed later when the time constraints imposed by the experiment are no longer present. At that time, a more sophisticated statistical analysis method could be used.

Data reliability is affected by experimental events such as intermittent electrical connections, sample motion in a magnetic field, rapid changes in temperature, periodic and random electrical noise, and movements of humans near a sensitive experiment. In addition, internally generated erroneous voltmeter readings have been observed. These particular errors scale with one of the following parameters: the bias level, the selected voltage range of the voltmeter, or the difference between the maximum allowable value of the selected range and the input signal. A typical error may be more than 100 times the standard deviation of the correct readings. Thus, a few erroneous readings can significantly affect the average of a collection of readings unless a data editor is in use. The fixed-limit editor has been developed to remove occasional erroneous readings that are inconsistent with the distribution of the majority of the readings. This editor is simple and expedient.

The software should give the user feedback on the experiment not only through data tables, but through audio and video signals as well. A system of this type could allow the experimenter to ascertain the source of an error, and possibly correct it. Unless a data editor is implemented in a data acquisition software routine, it is difficult to determine whether or not errors have occurred. High precision data can be obtained using an editor in the presence of outlying readings.

## Figures and Tables

**Figure 6a:**
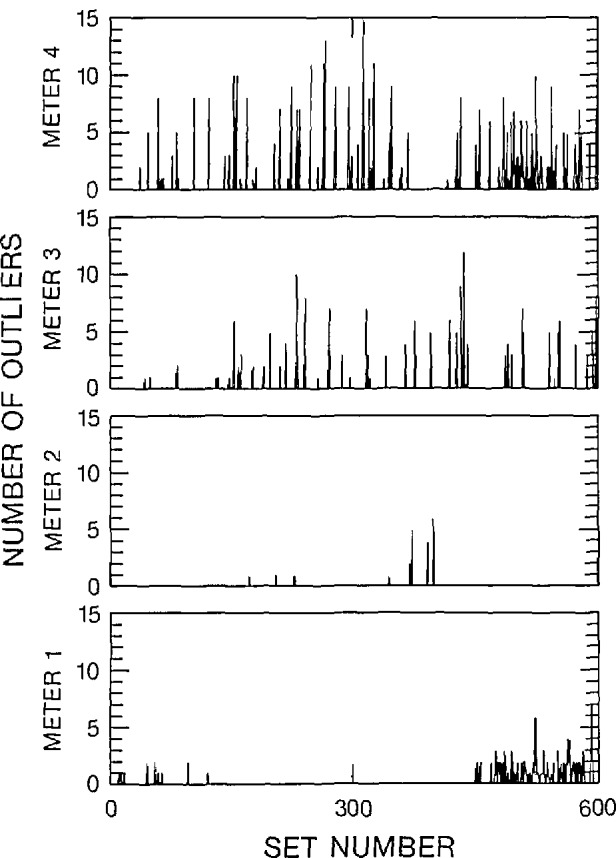
Number of outliers as a function of data set number, 2-V range, filter off.

**Figure 6b:**
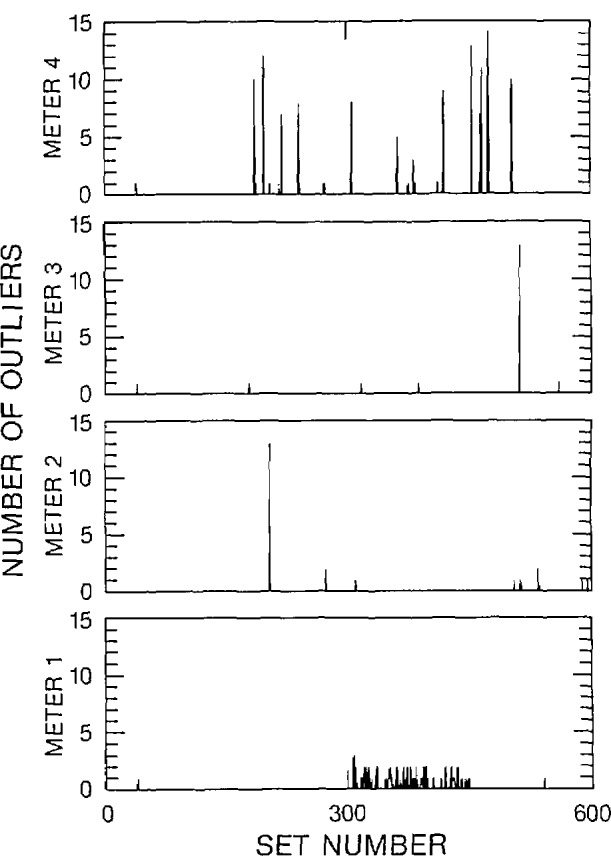
Number of outliers as a function of data set number, 2-mV range, filter off.

**Figure 7a:**
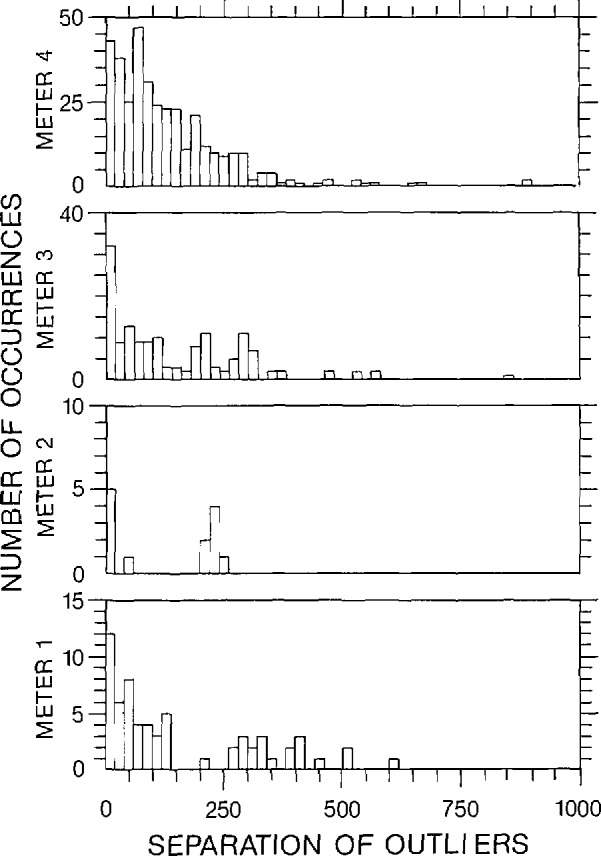
Number of outliers as a function of outliers separation, 2-V range, filter off.

**Figure 7b:**
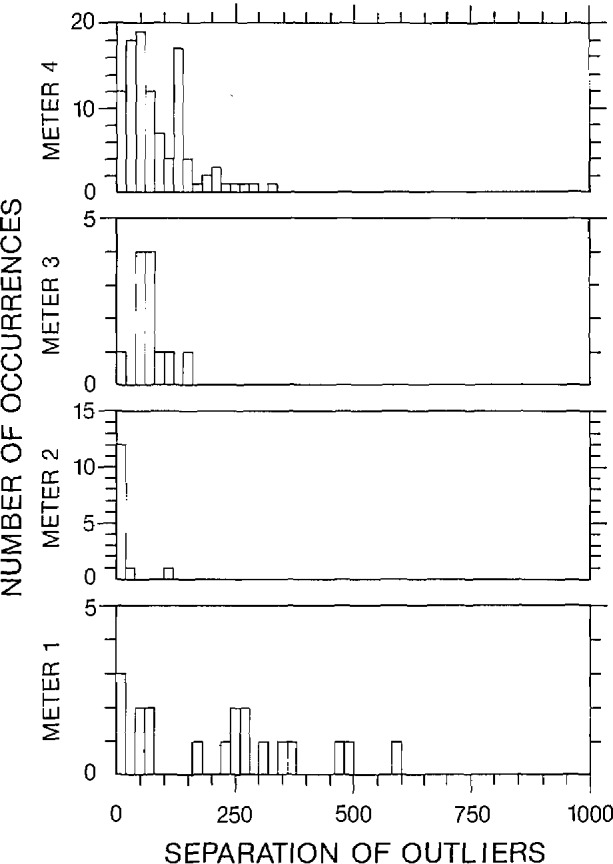
Number of outliers as a function of outlier separation, 2-mV range, filter off.

**Figure 8a:**
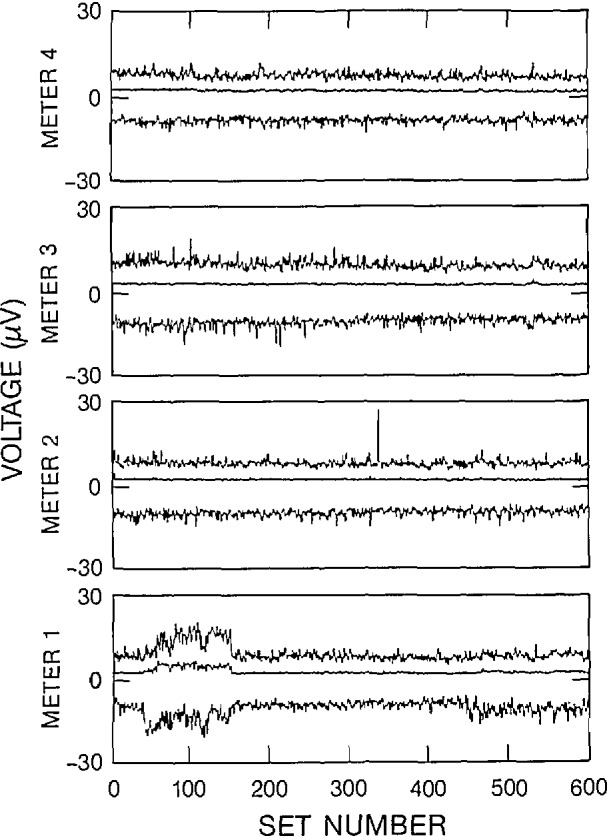
Edited voltage reading as a function of data set number, 2-V range, filter off.

**Figure 8b:**
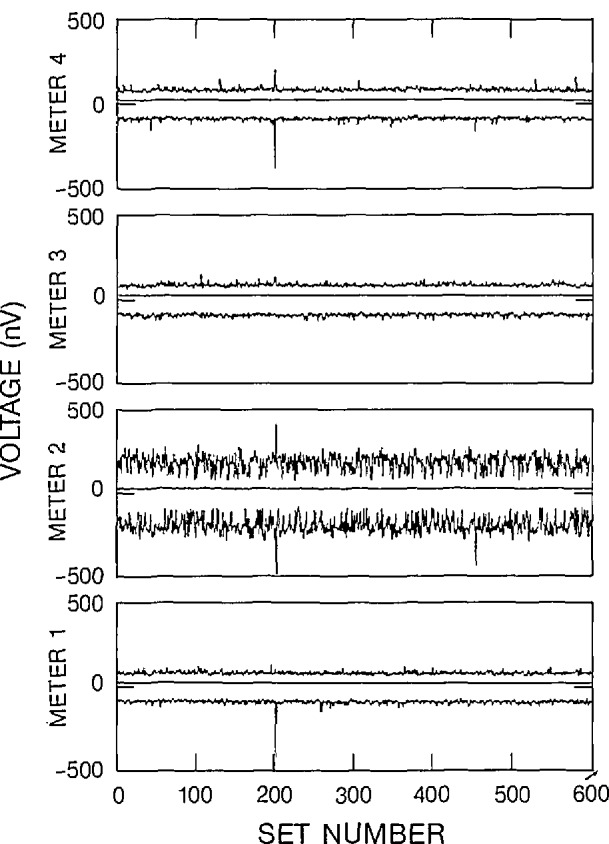
Edited voltage reading as a function of data set number, 2-mV range, filter off.

**Table 1a:** Percentage errors (number of occurrences in parentheses) for the 2-mV range, 4 bias levels, no filter

Meter	Bias, mV	% of bias		% of range	% of balance
		+0.6	−3.0	+3.1	−3.2
1	0.0				−3.051(1)
	0.2				
	0.6	0.560 (33)	−2.969 (35)		
	1.5		−2.968 (1)		
2	0.0				
	0.2				−3.278 (2)
	0.6	0.684 (1)			
	1.5	0.695 (2)	−3.098 (1)	3.200 (3)	
3	0.0				−3.066(1)
	0.2		−2.972 (1)		
	0.6	0.572 (1)	−2.971 (1)		
	1.5	0.565 (6)	−2.971 (8)		
4	0.0			3.097 (1)	
	0.2	0.597 (18)	−3.004(18)	3.101 (1)	−3.166 (1)
	0.6	0.597 (14)	−2.999 (12)	3.097 (1)	−3.349(1)
	1.5	0.595 (23)	−3.000(32)		

Notes: 234 outlyers for this data set. 15 points did not fit in a pattern.

**Table 1b:** Percentage errors (number of occurrences in parentheses) for the 20-mV range, 4 bias levels, no filter

Meter	Bias, mV	% of bias		% of range +	% of balance
		+0.6	−3.0	3.1	−3.2
1	0.0				
	0.2	0.558 (34)	−2.977(39)		
	0.6	0.562 (23)	−2.969(44)		
	1.5	0.563 (1)	−3.000 (2)		
2	0.0				
	0.2				−3.204(1)
	0.6		−3.088 (1)		
	1.5				
3	0.0				
	0.2	0.571 (12)	−2.961 (18)		
	0.6	0.569 (3)	−2.968 (7)		−3.082 (1)
	1.5	0.567 (23)	−2.971 (21)		
4	0.0				
	0.2	0.600 (4)	−2.985 (5)		
	0.6	0.592 (12)	−2.997 (13)		−3.116(1)
	1.5	0.592 (27)	−2.997 (33)	3.097 (1)	

Notes: 326 outlyers for this data set.

**Table 2a:** Percentage errors (number of occurrences in parentheses) for the 2-V range, 4 bias levels, no filter

Meter	Bias, V	% of bias	% of balance	
		0.6	+ 3.0	−3.1
1	0.0		2.966 (13)	
	0.2			
	0.6			
	1.5	0.561 (65)	2.967 (65)	
2	0.0			
	0.2	0.696 (2)	3.092 (1)	
	0.6	0.696 (8)	3.092 (10)	
	1.5			
3	0.0		2.972 (6)	−3.063 (1)
	0.2	0.567 (35)	2.972 (22)	−3.126(3)
	0.6	0.566 (27)	2.972 (43)	−3.304 (2)
	1.5	0.566 (43)	3.000 (16)	
4	0.0		2.999 (46)	−3.092 (3)
	0.2	0.595 (70)	2.999 (67)	−3.157(1)
	0.6	0.596 (46)	2.999 (36)	−3.346 (1)
	1.5	0.596 (106)	3.000 (85)	

Notes: 826 outlyers for this data set. 3 points did not fit in a pattern.

**Table 2b:** Percentage errors (number of occurrences in parentheses) for the 20-V range, 4 bias levels, no filter

Meter	Bias, V	% of bias	% of balance	
		0.6	+ 3.0	−3.1
1	0.0		2.966 (70)	
	0.2	0.557 (41)	2.966 (33)	
	0.6			
	1.5	0.559 (1)		
2	0.0			
	0.2	0.693 (3)		
	0.6	0.694 (9)	3.090 (14)	
	1.5	0.695 (53)	3.091 (49)	
3	0.0		2.972 (19)	
	0.2	0.565 (20)	2.972 (22)	
	0.6	0.566 (32)	2.972 (25)	
	1.5	0.566 (63)	2.972 (45)	
4	0.0		2.998 (32)	
	0.2	0.597 (43)	2.998 (37)	−3.097 (1)
	0.6	0.595 (66)	2.998 (55)	
	1.5	0.595 (21)	2.998 (20)	

Notes: 776 outlyers for this data set. 2 points did not fit in a pattern.

**Table 3a:** Error values in *µ*V, and the number of standard deviations above the edited readings (in parentheses) for the 2-mV range on meter 4, using a typical standard deviation of 28 nV for the edited readings

Bias, mV	% of bias		% of range	% of balance
	+0.6	−3.0	+3.1	−3.2
0.0			61.9 (2210)	−65.2^a^ (2330)
0.2	1.19 (43)	−6.0 (214)	62.0 (2210)	−57.0 (2040)
0.6	3.58 (128)	−18.0 (643)	61.9 (2210)	−46.8 (1670)
1.5	8.9 (318)	−45.0 (1610)	62.0^a^ (2210)	−16.3^a^ (582)

a These errors were not observed in the experiment.

**Table 3b:** Error values in mV, and the number of standard deviations above the edited readings (in parentheses) for the 2-V range on meter 4, using a typical standard deviation of 3 *µ*V for the edited readings

Bias, V	% of bias	% of balance	
	0.6	+3.0	−3.1
0.0		60.0 (20000)	−78.4 (26100)
0.2	1.19 (397)	54.0 (18000)	−56.8 (18900)
0.6	3.58 (1200)	42.0 (14000)	−46.8 (15600)
1.5	8.94 (2980)	15.0 (5000)	−16.0^a^ (5300)

a This error was not observed in the experiment.

## Footnotes

1 Certain commercial equipment, instruments, or materials mentioned in this paper might be indirectly identified by their particular properties. Such identification does not imply recommendation or endorsement by the National Institute of Standards and Technology, nor does it imply that the materials or equipment identified are necessarily the best available for the purpose.
